# From Nutritional Epidemiology to Clinical Prediction: Interpretable Machine Learning Reveals Subtle Associations Between Dietary Antioxidant Profiles and Non‐Melanoma Skin Cancer in Older Adults

**DOI:** 10.1002/fsn3.72458

**Published:** 2026-10-20

**Authors:** Bo Han Yu, Ji Young Kang, Kyung Eun Kim

**Affiliations:** ^1^ Department of Health Industry Sookmyung Women's University Seoul Republic of Korea

**Keywords:** aged, dietary antioxidant, machine learning, non‐melanoma skin cancer, predictive value of tests

## Abstract

The growing burden of non‐melanoma skin cancer (NMSC) among older adults underscores the need for early risk prediction based on modifiable dietary factors. In this cross‐sectional study using data from the 2007–2010 and 2017–2018 cycles of the National Health and Nutrition Examination Survey (NHANES), we evaluated dietary antioxidant profiles and developed an interpretable machine learning model for NMSC risk prediction. Baseline feature analysis revealed that participants with NMSC generally had higher socioeconomic status and higher oxidative balance scores (OBS), with significant differences also observed in age and race. Through feature importance ranking using the Random Forest (RF) algorithm, caffeine and luteolin were identified as the most predictive dietary variables. After comparing 12 machine learning algorithms, linear discriminant analysis (LDA) was selected as the final screening‐oriented prediction model (AUC = 0.742). Although its overall accuracy was lower than that of several ensemble models, LDA demonstrated higher sensitivity (0.730) and favorable imbalance‐aware performance, thereby mitigating the “accuracy paradox” and supporting its use for preliminary screening to identify participants with higher predicted NMSC risk. SHapley Additive exPlanations (SHAP) global explanations further confirmed that, after controlling for age as a primary risk factor, niacin and quercetin act as protective factors in the NMSC risk prediction model. In summary, this study developed an interpretable and robust machine learning model for NMSC risk prediction in older adults and provides hypothesis‐generating evidence regarding the potential role of dietary antioxidant profiles in prevention‐oriented strategies.

## Graphical abstract

This study was based on NHANES data. Older adults aged ≥ 60 years with available information on non‐melanoma skin cancer (NMSC), dietary intake, and covariates were included. Dietary antioxidant exposures were assessed using 24‐h dietary recall data, and demographic, socioeconomic, lifestyle, and health‐related variables were collected as covariates. After data preprocessing, feature selection was performed using LASSO, followed by the development and comparison of 12 machine‐learning models for NMSC prediction. Model performance was evaluated using multiple metrics, and SHAP analysis identified age, luteolin, caffeine, niacin, and quercetin as important predictive features, highlighting subtle associations between dietary antioxidant profiles and prevalent NMSC in older adults.

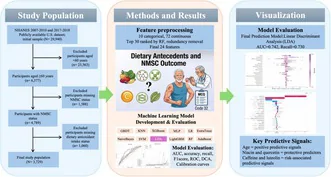

**Abbreviations**
AdaBoost: adaptive boosting
ANOVA: analysis of variance
AUC: area under the ROC curve
BMI: body mass index
CDAI: composite dietary antioxidant index
CIs: 95% confidence intervals
Cu: copper
DII: dietary inflammatory index
DNA: deoxyribonucleic acid
GBDT: gradient boosting decision tree
HEI‐2015: healthy eating index‐2015
KNN: kernel k‐nearest neighbors
LASSO: least absolute shrinkage and selection operator
LDA: linear discriminant analysis
LightGBM: light gradient boosting machine
LR: logistic regression
MCC: Matthews correlation coefficient
ML: machine learning
MLP: multilayer perceptron
MUFA: monounsaturated fatty acids
NaiveBayes: Gaussian naïve Bayes
NHANES: National Health and Nutrition Examination Survey
NMSC: non‐melanoma skin cancer
OBS: Oxidative Balance Score
PIR: poverty income ratio
PPV: positive predictive value
PUFA: polyunsaturated fatty acids
RF: random forest
ROC: receiver operating characteristic
SHAP: SHapley Additive exPlanations
SMOTE: synthetic minority oversampling technique
SVM: support vector machine
UV: ultraviolet
XGBoost: extreme gradient boosting
Zn: zinc

## 1 Introduction

Skin cancer is broadly classified into melanoma and non‐melanoma skin cancer (NMSC), with NMSC ranking as the fifth most commonly diagnosed cancer worldwide and imposing a growing public health burden (Hasan et al. 2023; Wang et al. 2025). According to  GLOBOCAN 2022 estimates, NMSC accounted for approximately 1.14 million new cases and 58,721 deaths worldwide in 2024 (Ervik et al. 2026). This burden is disproportionately concentrated in fair‐skinned populations and individuals aged 60 years or older, and ultraviolet (UV) radiation is a major established etiological factor for NMSC (Ali and Lear 2013; Wheless et al. 2010). Beyond UV exposure, however, increasing evidence indicates that modifiable factors, including dietary patterns, antioxidant intake, and lifestyle behaviors (Katta and Brown 2015; Skroza et al. 2020; Hezaveh et al. 2023), may contribute to interindividual differences in susceptibility. Accordingly, the identification of biologically plausible, accessible, and modifiable risk factors is important for strengthening NMSC prevention and risk prediction.

Antioxidants have been proposed as modifiable factors that may influence susceptibility to NMSC (Hezaveh et al. 2023). It can influence redox balance, inflammatory signaling, and immune regulation, they may be biologically relevant to NMSC‐related risk patterns. Several flavonoids, including myricetin and kaempferol have been reported to attenuate UV‐induced carcinogenic signaling in experimental models (Huang et al. 2010; Yao et al. 2014). Anthocyanins may mitigate ultraviolet B (UV‐B)‐induced reactive oxygen species generation, deoxyribonucleic acid (DNA) damage (Pratheeshkumar et al. 2014), and inflammatory activation (Guo et al. 2024). Quercetin has also exhibited potential anticancer activity in melanoma cells, particularly through the inhibition of cell proliferation (Seo et al. 2024). Furthermore, in a Phase 3 randomized controlled trial of high‐risk individuals, oral nicotinamide reduced the rate of new NMSC by 23% (Chen et al. 2015). Mediterranean diet components and related agri‐food bioactive products may also influence cancer‐related pathways through antioxidant, anti‐inflammatory and immunomodulatory (Perrone et al. 2025). Consistent with these findings, a higher Composite Dietary Antioxidant Index (CDAI) and greater adherence to anti‐inflammatory dietary patterns have been associated with a significantly lower risk of certain cancer and a reduced incidence of melanoma (Feng et al. 2025; Fortes et al. 2024).

Nevertheless, evidence regarding other dietary factors remains heterogeneous. Alcohol intake has been positively associated with basal cell carcinoma risk (Hezaveh et al. 2023). Selenium supplementation did not consistently reduce NMSC incidence in randomized trial settings, and some antioxidant supplementation studies have suggested that the effects may depend on sex, supplement composition, and baseline risk status (Duffield‐Lillico et al. 2003; Hercberg et al. 2007). Zinc (Zn) also appears to influence cutaneous antioxidant defense and immune function in a dose‐ and context‐dependent manner (Altobelli et al. 2020). These discrepancies suggest that the relationship between dietary antioxidants and NMSC may not be explained by single nutrients alone but may instead depend on dose, bioavailability, dietary context, co‐occurring lifestyle factors, and population‐specific susceptibility.

Machine learning (ML) may complement conventional statistical methods by capturing high‐dimensional, nonlinear, and interactive patterns among co‐occurring dietary exposures (Morgenstern et al. 2021). SHAP is a useful and effective method for quantifying the marginal contribution of each feature to model output, thereby enhancing model interpretability (Guo et al. 2025). The NHANES (CDC 2026), which combines detailed dietary, demographic, laboratory test and health questionnaire data from a nationally representative sample, provides a suitable platform for investigating the nutritional determinants of NMSC. Recent studies based on NHANES data have examined individual nutrients or composite antioxidant indices in relation to cancer and skin cancer outcomes, including vitamin B1 intake and NMSC risk (Huang et al. 2025) and CDAI in cancer‐related outcomes (Feng et al. 2025). However, these studies have focused on individual nutrients, broad cancer outcomes, or non‐NMSC‐specific prediction tasks. In contrast, daily antioxidant exposure reflects a complex mixture of bioactive compounds rather than isolated nutrients. To our knowledge, NMSC‐specific risk prediction based on multiple dietary antioxidants, particularly among older adults, has not been comprehensively examined, leaving a gap in the current literature.

Therefore, limited evidence has systematically integrated NHANES‐derived multiple dietary antioxidants with interpretable ML to predict and explain NMSC risk among older adults. This study, based on a cross‐sectional design, uses an interpretable, data‐driven approach to fill the gaps in single‐nutrient analysis and conventional regression frameworks. It may contribute to the identification of NMSC risk factors and help generate hypotheses for targeted nutritional prevention strategies in older populations.

## 2 Materials and Methods

### 2.1 Data Source and Study Population

Data were derived from the 2007–2010 and 2017–2018 cycles of the NHANES survey, comprising 29,940 participants. These cycles were selected because they provided both the cancer questionnaire data required to ascertain the outcome and detailed 24‐h dietary recall data needed to assess dietary exposure, and referenced recent nutritional studies based on NHANES (Tu et al. 2026; Xu et al. 2026). The 2011–2016 cycles were not included because the required outcome and dietary exposure variables were not simultaneously available. Participants younger than 60 years were excluded to focus on older adults, among whom NMSC risk was considered more clinically relevant, and to maintain an adequate balance between NMSC and non‐NMSC participants. Eligible participants were required to have available information on NMSC status and complete dietary antioxidant intake data. The detailed participant selection process are shown in Figure 1.

**FIGURE 1:**
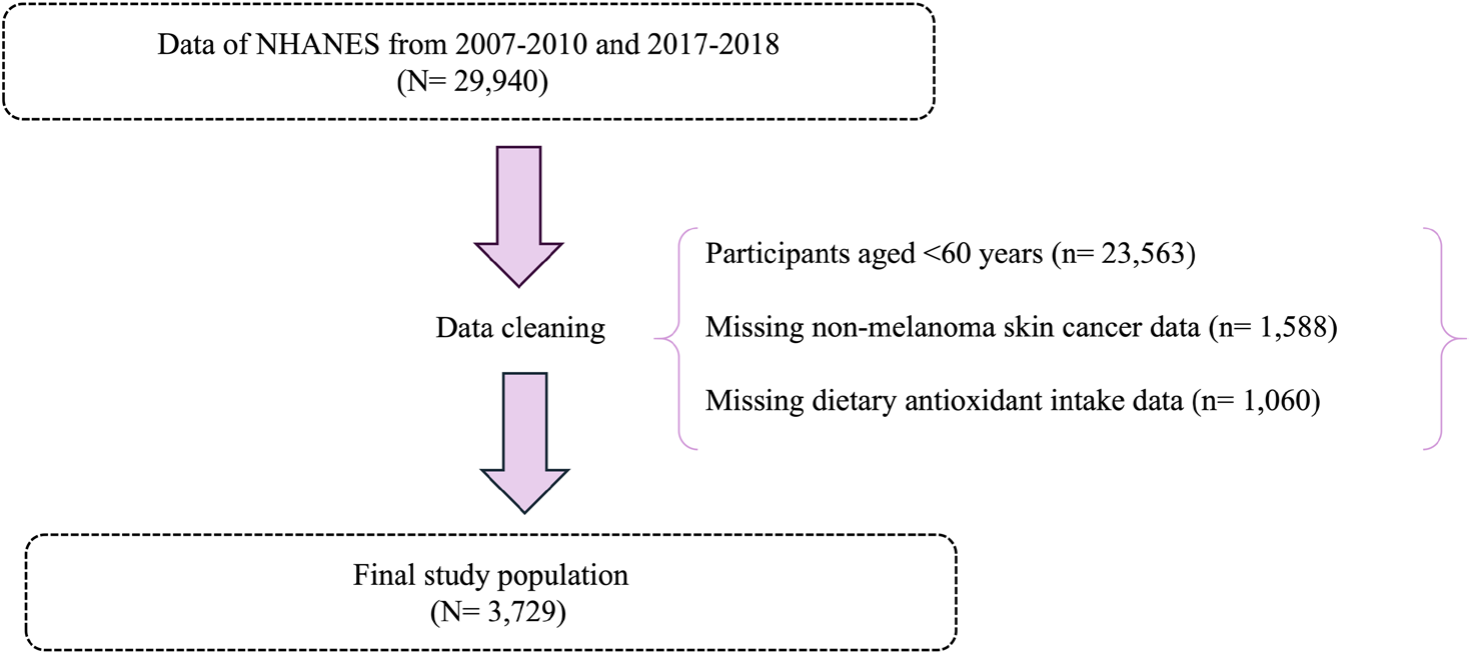
Participants screening flow chart. Individuals younger than 60 years, those without information on non‐melanoma skin cancer status, and those with incomplete dietary antioxidant intake data were excluded. The final analytic sample included participants with complete outcome, dietary exposure, and covariate information.

### 2.2 Assessment of Non‐Melanoma Skin Cancer

Information on NMSC was obtained from the Medical Conditions section of the NHANES questionnaire. Participants were first asked whether a doctor or another healthcare professional had informed them that they had cancer. Those who answered affirmatively were asked to report the specific type of cancer. In the NHANES dataset, code “32” corresponds to “NMSC” and was defined as the target outcome in this study. For analysis, participants with code “32” were classified as having NMSC and coded as “1”, whereas all other participants were coded as “0”. NHANES does not provide histopathological confirmation or subtype information for this questionnaire item; the outcome in this study represents self‐reported overall NMSC status.

### 2.3 Assessment of Dietary Antioxidant and Other Nutrient Intake

Dietary intake data encompassing macronutrients, micronutrients, flavonoid subclasses, and caffeine were derived from the NHANES total nutrient intake files (DR2TOT_E, DR2TOT_F and DR2TOT_J). Dietary information was collected through two 24‐h dietary recall interviews conducted at mobile examination centers and administered 3–10 days apart. The average daily intake for each variable was calculated across both recalls. To comprehensively characterize the dietary antioxidant exposure profile and its potential relevance to NMSC risk, four composite dietary indices were constructed: Composite Dietary Antioxidant Index (CDAI), Oxidative Balance Score (OBS), Healthy Eating Index‐2015 (HEI‐2015), and Dietary Inflammatory Index (DII). Detailed calculation procedures are provided in Table S1.

### 2.4 Collection of Baseline Features

Baseline characteristics were collected from the demographic, socioeconomic, anthropometric, and lifestyle domains. Demographic variables included age (continuous), sex (male/female), race/ethnicity (Non‐Hispanic White, Non‐Hispanic Black, Mexican American, Other Hispanic, and other races/multiracial), marital status (married/cohabiting, widowed/divorced/separated, and never married), and educational attainment (less than high school, high school graduate, and above high school). Socioeconomic status was indexed by the poverty income ratio (PIR) and categorized as < 1, 1–3, or > 3. Anthropometric assessments were based on body mass index (BMI). Lifestyle and behavioral factors included smoking status (never, former, and current smoker) and alcohol consumption (estimated daily intake in grams derived from 24‐h dietary recalls).

### 2.5 Preprocessing of Machine Learning Features

The initial analytical dataset comprised 82 candidate features, including 72 continuous and 10 categorical variables. Before imputation, the number and percentage of missing values were calculated for each candidate feature in Table S2. Missing data were addressed using missForest, a nonparametric iterative imputation algorithm based on RF that accommodates mixed‐type data without distributional assumptions. To identify an appropriate feature optimization framework, logistic regression (LR), Least absolute shrinkage and selection operator (LASSO), and RF were compared based on receiver operating characteristic (ROC) curves and area under the ROC curve (AUC) values. Pearson correlation analysis was subsequently applied to reduce redundancy among candidate features, with variables exhibiting pairwise correlations of |*r*| > 0.7 excluded to mitigate multicollinearity and improve model robustness. To address the substantial class imbalance between NMSC cases and controls, the synthetic minority oversampling technique (SMOTE) was applied exclusively to the training set after the initial train‐test split. The held‐out test set was not subjected to SMOTE, oversampling, undersampling, or any other resampling procedure. Furthermore, we reduced majority class bias during model training by generating synthetic minority‐class samples by interpolation between K‐nearest neighbors.

### 2.6 Statistical Analysis

Baseline characteristics were summarized using survey‐weighted methods to account for the complex sampling design of NHANES. Continuous variables were presented as mean ± standard error (SE), and categorical variables as weighted frequencies and proportions. Between‐group differences in categorical variables were evaluated using weighted chi‐squared tests, whereas weighted analysis of variance (ANOVA) and Kruskal–Wallis tests were applied to normally distributed and non‐normally distributed continuous variables, respectively. To evaluate and compare predictive performance, 12 machine learning algorithms were developed: LR, support vector machine (SVM), RF, extreme gradient boosting (XGBoost), light gradient boosting machine (LightGBM), multilayer perceptron (MLP), kernel k‐nearest neighbors (KNN), Gaussian naïve Bayes (NaiveBayes), extra trees, adaptive boosting (AdaBoost), gradient boosting decision tree (GBDT), and linear discriminant analysis (LDA).

Model performance was assessed using the AUC with 95% confidence intervals (CIs), along with accuracy, precision/positive predictive value (PPV), sensitivity, and F1 score, we calculated specificity, balanced accuracy and Matthews correlation coefficient (MCC). Calibration curves were constructed to assess agreement between predicted probabilities and observed outcomes, and decision curve analysis was conducted to estimate the net clinical benefit across a range of threshold probabilities. All reported predictive performance metrics were calculated using the original held‐out test set, which remained completely independent of feature resampling and model training. The final screening‐oriented prediction model was selected based on overall discriminative performance and clinical utility; SHAP LinearExplainer was then used to quantify feature contributions at both global and individual prediction levels. These values were interpreted as model‐specific predictive contributions rather than causal effects.

All statistical analyses were performed using R (version 4.3.0). Statistical significance was set at *p* < 0.05 for all analyses.

## 3 Results

### 3.1 Population Characteristics

A total of 3729 older adults were included, comprising 3607 controls and 122 participants diagnosed with NMSC. Baseline characteristics are summarized in Table 1. The two groups differed significantly in demographic, socioeconomic, metabolic, and dietary characteristics. Participants in the NMSC group were significantly older and were predominantly non‐Hispanic White (94.3% vs. 48.6%, *p* < 0.001). Participants with NMSC also exhibited higher socioeconomic status, as reflected by a greater proportion of individuals with education above the high school level (63.1%, *p* < 0.001) and a PIR greater than 3 (51.6% vs. 36.8%, *p* < 0.001). The prevalence of diabetes was significantly lower in the NMSC group than in the non‐NMSC group (23.8% vs. 34.8%, *p* = 0.008). In terms of dietary and antioxidant profiles, the NMSC group had a higher OBS (20.56 ± 6.93 vs. 18.87 ± 7.09, *p* = 0.009) and a lower DII (1.08 ± 1.88 vs. 1.59 ± 1.85, *p* = 0.004). Intake of several micronutrients and flavonoids, including vitamin E, Zn, calcium, magnesium, and anthocyanidins, was significantly higher in the NMSC group (all *p* < 0.05). Additionally, current alcohol consumption was higher among participants in the NMSC group than among controls (*p* < 0.001).

**TABLE 1:** Baseline characteristics of participants.

Characteristic	Overall (*n* = 3729)	No non‐melanoma skin cancer (*n* = 3607)	Non‐melanoma skin cancer (*n* = 122)	*p*
Gender, *n* (%)				
Female	1824 (48.9)	1749 (48.5)	75 (61.5)	0.006
Male	1905 (51.1)	1858 (51.5)	47 (38.5)	
Race, *n* (%)				
Non‐Hispanic White	1869 (50.1)	1754 (48.6)	115 (94.3)	< 0.001
Non‐Hispanic Black	810 (21.7)	810 (22.5)	0 (0.0)	
Mexican American	472 (12.7)	467 (12.9)	5 (4.1)	
Other Hispanic	353 (9.5)	353 (9.8)	0 (0.0)	
Other Race	225 (6.0)	223 (6.2)	2 (1.6)	
Marital status, *n* (%)	3729 (100.00)	3607 (96.7)	122 (3.3)	0.511
Married/living with partner	2197 (58.9)	2122 (58.8)	75 (61.5)	
Widowed/divorced/separated	1363 (36.6)	1319 (36.6)	44 (36.1)	
Never married	169 (4.5)	166 (4.6)	3 (2.5)	
PIR, *n* (%)	3729 (100.00)	3607 (96.7)	122 (3.3)	< 0.001
< 1	870 (23.3)	857 (23.8)	13 (10.7)	
1–3	1470 (39.4)	1424 (39.5)	46 (37.7)	
> 3	1389 (37.2)	1326 (36.8)	63 (51.6)	
Education level, *n* (%)	3729 (100.00)	3607 (96.7)	122 (3.3)	< 0.001
Below high school	551 (14.8)	545 (15.1)	6 (4.9)	
High school	1445 (38.8)	1406 (39.0)	39 (32.0)	
Above high school	1733 (46.5)	1656 (45.9)	77 (63.1)	
Smoker, *n* (%)	3729 (100.00)	3607 (96.7)	122 (3.3)	0.008
Never	1813 (48.6)	1765 (48.9)	48 (39.3)	
Former	1459 (39.1)	1395 (38.7)	64 (52.5)	
Current	457 (12.3)	447 (12.4)	10 (8.2)	
Alcohol, *n* (%)	3729 (100.00)	3607 (96.7)	122 (3.3)	< 0.001
Never	615 (16.5)	608 (16.9)	7 (5.7)	
Former	731 (19.6)	717 (19.9)	14 (11.5)	
Current	2383 (63.9)	2282 (63.3)	101 (82.8)	
BMI, *n* (%)	3729 (100.00)	3607 (96.7)	122 (3.3)	0.913
< 18	822 (22.0)	797 (22.1)	25 (20.5)	
18–25	1358 (36.4)	1313 (36.4)	45 (36.9)	
> 25	1549 (41.5)	1497 (41.5)	52 (42.6)	
DM, *n* (%)	3729 (100.00)	3607 (96.7)	122 (3.3)	0.008
No	2444 (65.5)	2351 (65.2)	93 (76.2)	
Yes	1285 (34.5)	1256 (34.8)	29 (23.8)	
Stroke, *n* (%)	3729 (100.00)	3607 (96.7)	122 (3.3)	0.175
No	3403 (91.3)	3287 (91.1)	116 (95.1)	
Yes	326 (8.7)	320 (8.9)	6 (4.9)	
Age (year), mean ± SD	69.58 ± 6.89	69.45 ± 6.87	73.57 ± 6.27	< 0.001
Alpha_carotene (μg/day), mean ± SD	428.06 ± 819.14	425.59 ± 820.84	500.84 ± 766.94	0.29
Beta_carotene (μg/day), mean ± SD	2394.08 ± 3078.20	2383.73 ± 3079.25	2699.95 ± 3043.36	0.261
Vitamin_A_RAE (μg/day), mean ± SD	217.34 ± 279.42	216.38 ± 279.53	245.86 ± 275.64	0.248
VitA (μg/day), mean ± SD	636.99 ± 574.88	633.31 ± 573.07	745.81 ± 618.72	0.05
Vit (mg/day), mean ± SD	83.32 ± 71.25	83.29 ± 71.60	84.19 ± 60.13	0.872
VitE (mg/day), mean ± SD	7.22 ± 4.42	7.18 ± 4.40	8.51 ± 4.76	0.003
Riboflavin (mg/day), mean ± SD	1.88 ± 0.88	1.87 ± 0.88	2.17 ± 0.92	< 0.001
Niacin (mg/day), mean ± SD	21.48 ± 9.95	21.44 ± 9.97	22.67 ± 9.12	0.144
VitB6 (mg/day), mean ± SD	1.83 ± 1.05	1.83 ± 1.05	1.93 ± 0.84	0.231
Folate (μg/day), mean ± SD	357.87 ± 178.26	356.71 ± 178.55	392.17 ± 166.59	0.023
VitB12 (μg/day), mean ± SD	4.65 ± 5.04	4.63 ± 5.02	5.28 ± 5.48	0.198
Thiamin (mg/day), mean ± SD	1.44 ± 0.63	1.44 ± 0.63	1.58 ± 0.62	0.013
Calcium (mg/day), mean ± SD	821.05 ± 436.13	818.28 ± 437.22	902.93 ± 395.40	0.022
Magnesium (mg/day), mean ± SD	270.36 ± 114.19	269.67 ± 114.49	290.91 ± 103.48	0.028
Iron (mg/day), mean ± SD	13.61 ± 6.72	13.57 ± 6.73	14.68 ± 6.21	0.056
Zinc (mg/day), mean ± SD	10.04 ± 5.34	9.99 ± 5.34	11.29 ± 5.16	0.007
Copper (mg/day), mean ± SD	1.17 ± 0.85	1.17 ± 0.84	1.29 ± 0.96	0.173
Selenium (μg/day), mean ± SD	97.55 ± 43.98	97.47 ± 44.13	100.04 ± 39.32	0.482
CDAI (score), mean ± SD	0.02 ± 3.58	−0.00 ± 3.58	0.70 ± 3.54	0.033
DII (score), mean ± SD	1.57 ± 1.86	1.59 ± 1.85	1.08 ± 1.88	0.004
HEI‐2015 (score), mean ± SD	56.94 ± 13.24	56.96 ± 13.26	56.47 ± 12.61	0.674
OBS (score), mean ± SD	18.92 ± 7.09	18.87 ± 7.09	20.56 ± 6.93	0.009
Daidzei (mg/day), mean ± SD	0.58 ± 4.37	0.56 ± 4.28	1.20 ± 6.50	0.284
Genistein (mg/day), mean ± SD	0.79 ± 5.97	0.76 ± 5.85	1.61 ± 8.79	0.291
Glycitein (mg/day), mean ± SD	0.11 ± 0.98	0.10 ± 0.96	0.27 ± 1.51	0.238
Cyanidin (mg/day), mean ± SD	2.67 ± 11.32	2.63 ± 11.24	4.08 ± 13.53	0.243
Petunidin (mg/day), mean ± SD	1.10 ± 5.07	1.02 ± 4.87	3.27 ± 9.00	0.007
Delphinidin (mg/day), mean ± SD	1.50 ± 7.08	1.42 ± 6.94	3.70 ± 10.15	0.015
Malvidin (mg/day), mean ± SD	4.63 ± 15.81	4.45 ± 15.52	10.22 ± 22.11	0.005
Pelargonidin (mg/day), mean ± SD	1.54 ± 6.34	1.51 ± 6.26	2.32 ± 8.27	0.291
Peonidin (mg/day), mean ± SD	1.71 ± 9.25	1.65 ± 9.25	3.61 ± 9.23	0.023
Catechin (mg/day), mean ± SD	7.49 ± 9.48	7.47 ± 9.52	7.96 ± 8.16	0.518
Epigallocatechin (mg/day), mean ± SD	15.10 ± 43.77	15.18 ± 44.13	12.65 ± 31.49	0.391
Epicatechin (mg/day), mean ± SD	9.37 ± 14.61	9.39 ± 14.73	8.87 ± 10.49	0.595
Epicatechin_3_gallate (mg/day), mean ± SD	9.63 ± 28.05	9.68 ± 28.28	8.08 ± 20.33	0.4
Epigallocatechin_3_gallate (mg/day), mean ± SD	26.42 ± 90.22	26.56 ± 90.92	22.32 ± 66.53	0.496
Theaflavins (mg/day), mean ± SD	1.35 ± 3.85	1.35 ± 3.88	1.15 ± 2.76	0.422
Thearubigins (mg/day), mean ± SD	79.92 ± 208.66	80.22 ± 209.98	70.90 ± 165.41	0.545
Eriodictyol (mg/day), mean ± SD	0.15 ± 0.74	0.14 ± 0.73	0.20 ± 0.83	0.494
Hesperetin (mg/day), mean ± SD	10.40 ± 23.63	10.41 ± 23.74	9.89 ± 20.01	0.779
Naringenin (mg/day), mean ± SD	4.35 ± 12.52	4.36 ± 12.62	4.06 ± 8.81	0.718
Apigenin (mg/day), mean ± SD	0.22 ± 2.32	0.22 ± 2.36	0.16 ± 0.27	0.199
Luteolin (mg/day), mean ± SD	0.63 ± 1.04	0.62 ± 1.02	0.84 ± 1.51	0.113
Isorhamnetin (mg/day), mean ± SD	0.78 ± 1.44	0.78 ± 1.43	0.87 ± 1.62	0.538
Kaempferol (mg/day), mean ± SD	3.96 ± 5.87	3.97 ± 5.91	3.48 ± 4.29	0.22
Myricetin (mg/day), mean ± SD	1.48 ± 2.60	1.48 ± 2.61	1.60 ± 2.19	0.545
Quercetin (mg/day), mean ± SD	10.59 ± 10.87	10.59 ± 10.87	10.75 ± 10.77	0.871
Theaflavin_3_3_digallate (mg/day), mean ± SD	1.49 ± 4.26	1.49 ± 4.29	1.27 ± 3.06	0.439
Theaflavin_3q_gallate (mg/day), mean ± SD	1.24 ± 3.66	1.25 ± 3.69	1.04 ± 2.58	0.374
Theaflavin_3_gallate (mg/day), mean ± SD	1.07 ± 3.06	1.08 ± 3.08	0.94 ± 2.24	0.496
Gallocatechin (mg/day), mean ± SD	1.44 ± 3.84	1.45 ± 3.87	1.21 ± 2.75	0.342
Subtotal_Catechins (mg/day), mean ± SD	69.46 ± 183.79	69.74 ± 185.25	61.09 ± 133.99	0.49
Isoflavones (mg/day), mean ± SD	1.48 ± 11.28	1.43 ± 11.05	3.08 ± 16.79	0.283
Anthocyanidins (mg/day), mean ± SD	13.16 ± 36.40	12.68 ± 35.53	27.20 ± 54.87	0.004
Flavan_3_ols (mg/day), mean ± SD	154.53 ± 364.44	155.14 ± 367.26	136.37 ± 268.52	0.455
Flavanones (mg/day), mean ± SD	14.89 ± 32.42	14.92 ± 32.59	14.15 ± 26.81	0.758
Flavones (mg/day), mean ± SD	0.85 ± 2.66	0.84 ± 2.69	1.00 ± 1.62	0.297
Flavonols (mg/day), mean ± SD	16.82 ± 18.18	16.82 ± 18.23	16.70 ± 16.74	0.94
Total_Sum_of_all_29_flavonoids (mg/day), mean ± SD	201.72 ± 385.01	201.83 ± 387.72	198.51 ± 295.34	0.904
Fiber (g/day), mean ± SD	16.18 ± 8.19	16.14 ± 8.20	17.43 ± 7.70	0.072
Total_Fat (g/day), mean ± SD	68.69 ± 32.62	68.45 ± 32.68	76.02 ± 30.19	0.007
Alcohol (g/day), mean ± SD	5.40 ± 15.31	5.25 ± 15.19	9.85 ± 18.14	0.006
Cotinine (ng/mL), mean ± SD	43.47 ± 118.72	43.71 ± 119.14	36.36 ± 105.76	0.454
Caffeine (g/day), mean ± SD	0.15 ± 0.16	0.15 ± 0.16	0.20 ± 0.19	0.003
Cholesterol (mg/day), mean ± SD	254.31 ± 144.78	254.19 ± 145.00	258.02 ± 138.84	0.765
Mufa (g/day), mean ± SD	24.26 ± 11.40	24.18 ± 11.41	26.71 ± 10.88	0.013
N6_fatty_acids (g/day), mean ± SD	15.46 ± 8.27	15.42 ± 8.29	16.76 ± 7.55	0.056
Protein (g/day), mean ± SD	70.16 ± 28.35	70.05 ± 28.45	73.45 ± 25.33	0.148
Pufa (g/day), mean ± SD	15.40 ± 7.47	15.35 ± 7.48	16.75 ± 7.03	0.032
Saturated_fat (g/day), mean ± SD	21.99 ± 11.56	21.87 ± 11.51	25.36 ± 12.37	0.003

### 3.2 Preliminary Model Selection

Our initial variable selection aimed to reduce feature dimensionality, remove redundant or noisy variables, and improve the stability and interpretability of subsequent ML models. LASSO regression was first applied for preliminary variable selection. Cross‐validation identified the optimal penalty parameter at which the binomial deviance was minimized, supporting the stability of LASSO‐based shrinkage and dimensionality reduction (Figure S1). The three candidate methods exhibited distinct feature‐prioritization profiles (Figure 2A).

**FIGURE 2:**
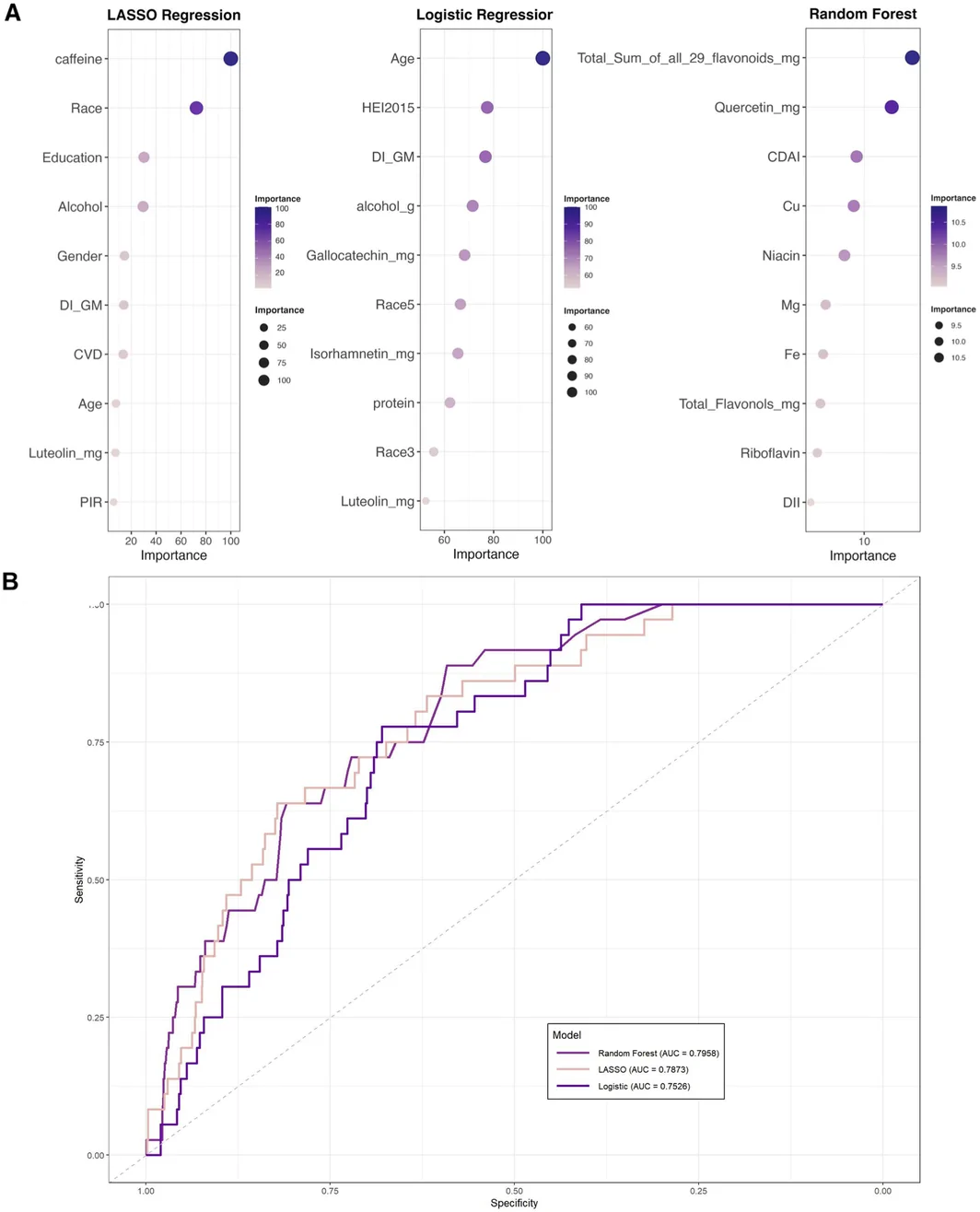
Preliminary comparison of three models for predicting NMSC risk. (A) Dot plots showing the top 10 features ranked by variable importance for LASSO regression, Logistic Regression (LR), and Random Forest (RF), with darker shades indicating higher importance. (B) ROC curves comparing the discriminative performance of the three models; the diagonal dashed line represents chance‐level discrimination. AUC, area under the ROC curve; LASSO, least absolute shrinkage and selection operator; ROC, receiver operating characteristic.

LASSO was dominated by caffeine and sociodemographic variables, LR regression by age and the HEI‐2015, LR regression by caffeine and sociodemographic variables, and RF by dietary antioxidant‐related features, including total flavonoids, quercetin, and CDAI. Discriminative performance was subsequently assessed using ROC curves (Figure 2B). RF achieved the highest AUC (0.7958), followed by LASSO regression and LR. Collectively, these findings indicated that RF demonstrated the strongest preliminary discriminative performance among the three approaches and was therefore selected for subsequent feature optimization and model construction.

### 3.3 Feature Selection and Machine Learning Model Development

Feature selection was performed in two sequential steps using a RF model. The Variable importance ranking based on the mean decrease in accuracy identified the top 30 predictors (Figure 3), most of which were diet‐related. Caffeine showed the highest importance score (5.84), followed by age, luteolin, saturated Fat, alpha‐carotene, and quercetin. Other highly ranked predictors included flavonoid compounds, antioxidant vitamins, micronutrients, and dietary quality indices, suggesting that nutritional factors constitute an important dimension in discriminating NMSC risk. Pearson correlation analysis was subsequently applied to reduce multicollinearity, and variables with an absolute correlation coefficient greater than 0.70 were excluded (Figures 4 and S2; Table S3). Before filtering, strong correlations were observed between quercetin and total flavonols (*r* = 0.96), total flavonols and total flavonoids (*r* = 0.84), and vitamin B6 and niacin (*r* = 0.80). After removing six redundant variables, namely, total flavonols, copper (Cu), total flavonoids, vitamin B12, vitamin B6, and thiamin, 24 relatively independent features were retained. These features included demographic characteristics, dietary fat intake, antioxidant‐related exposures, and flavonoid‐related domains.

**FIGURE 3:**
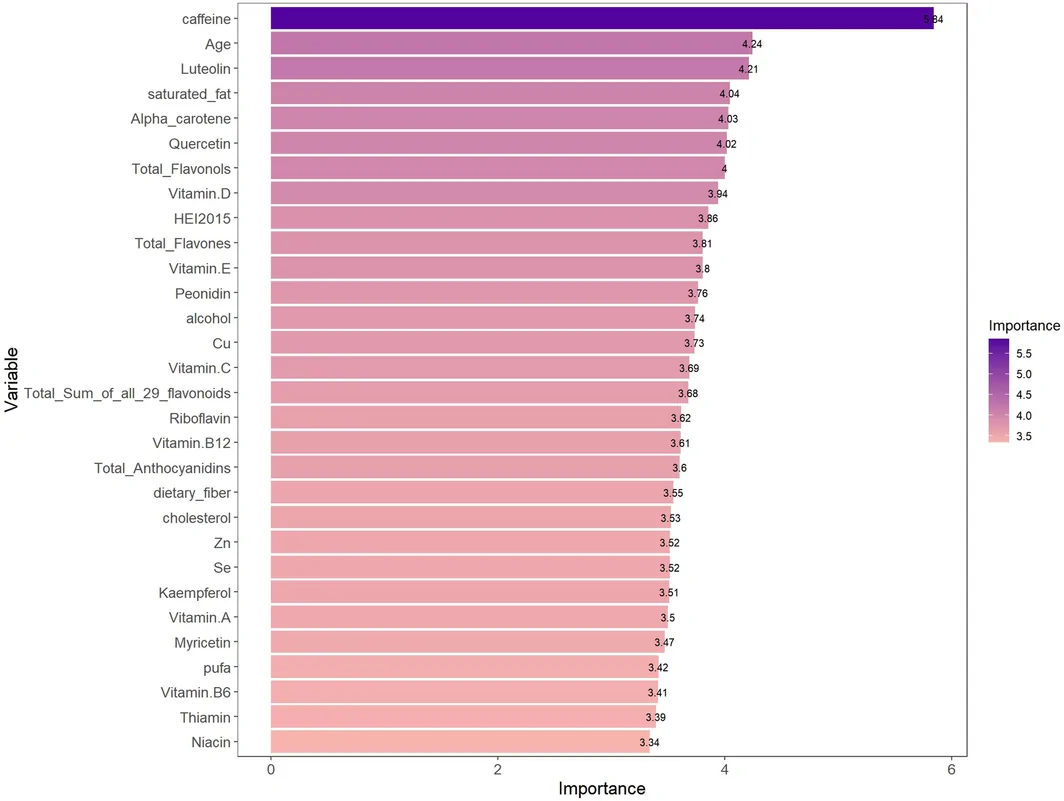
Random forest‐based feature importance ranking for non‐melanoma skin cancer prediction. The top 30 predictors were ranked according to the mean decrease in accuracy derived from the random forest model. Higher importance scores indicate a greater contribution to model discrimination.

**FIGURE 4:**
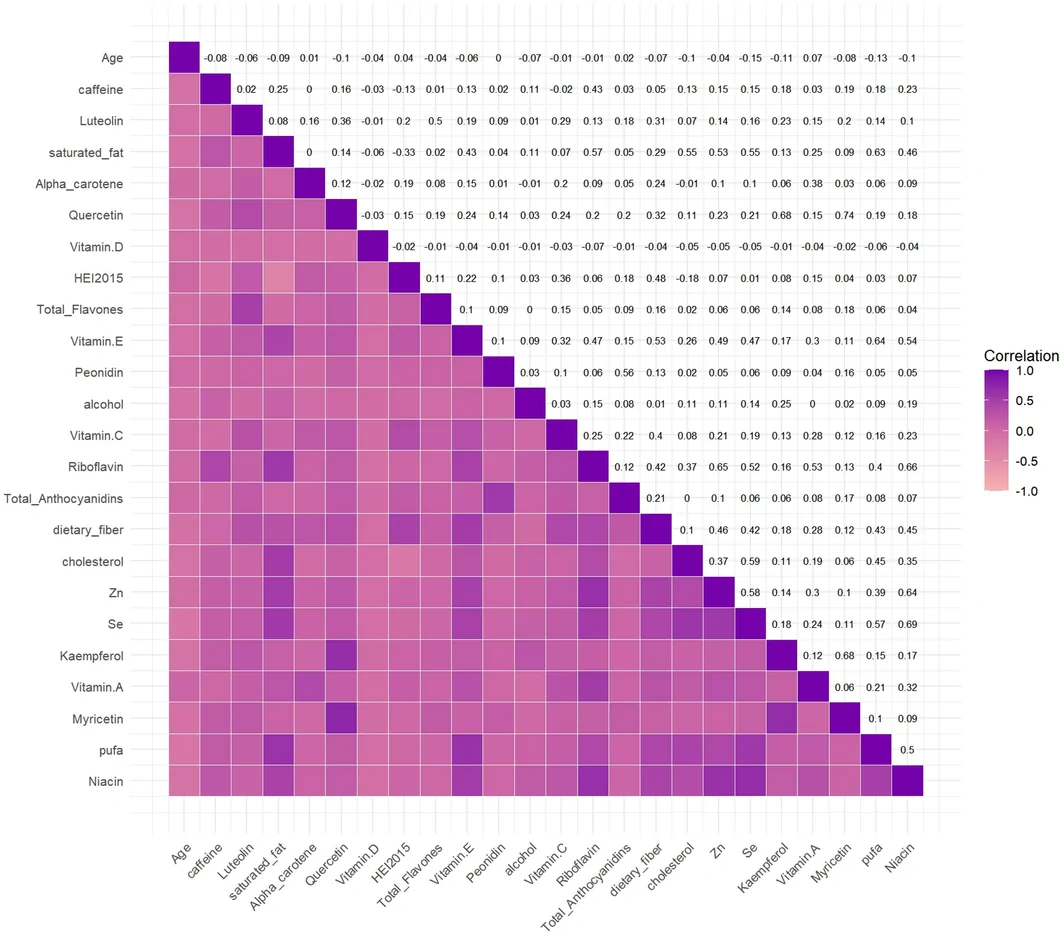
Pearson correlation matrix of candidate predictors after feature filtering. Pearson correlation analysis was performed to assess multicollinearity among candidate predictors selected from the random forest‐based feature importance ranking. The color scale represents the Pearson correlation coefficient, ranging from −1 to 1. Variables with an absolute correlation coefficient greater than 0.70 were considered highly correlated and were removed to reduce multicollinearity.

### 3.4 Final Risk Prediction Model Identification and Evaluation

We comprehensively evaluated 12 machine learning algorithms using the test set. Although several ensemble methods, such as ExtraTrees and RF, achieved a high overall accuracy of greater than 0.93, their sensitivity was limited (0.081 and 0.135), indicating that most NMSC cases were missed. LDA and LR achieved the highest AUC values, both reaching 0.742. Compared with LR, LDA showed slightly higher sensitivity (0.730 vs. 0.703), balanced accuracy (0.703 vs. 0.695), and MCC (0.153 vs. 0.149) in Table 2 and Figures 5 and 6. Model performance was assessed using AUC (Figure 6A), Precision/PPV (Figure 6B), Sensitivity (Figure 6C), Specificity (Figure 6D), Balanced accuracy (Figure 6E) and MCC (Figure 6F).

**TABLE 2:** Performance comparison of 12 machine learning models in the test set.

Model	AUC (95% CIs)	Accuracy	Precision/ppv	Sensitivity	Specificity	Balanced accuracy	MCC	F1 Score
LR	0.742 (0.668–0.815)	0.688 (0.662–0.715)	0.071 (0.047–0.099)	0.703 (0.552–0.849)	0.688 (0.661–0.714)	0.695 (0.618–0.769)	0.149 (0.089–0.210)	0.130 (0.087–0.175)
LDA	0.742 (0.666–0.815)	0.677 (0.651–0.704)	0.071 (0.047–0.098)	0.730 (0.583–0.862)	0.676 (0.649–0.702)	0.703 (0.626–0.770)	0.153 (0.093–0.212)	0.130 (0.088–0.174)
AdaBoost	0.666 (0.587–0.749)	0.783 (0.761–0.808)	0.064 (0.036–0.098)	0.405 (0.256–0.586)	0.796 (0.773–0.820)	0.601 (0.525–0.690)	0.088 (0.023–0.161)	0.110 (0.062–0.165)
ExtraTrees	0.640 (0.556–0.725)	0.959 (0.947–0.971)	0.200 (0.000–0.417)	0.081 (0.000–0.182)	0.989 (0.982–0.994)	0.535 (0.496–0.585)	0.109 (−0.016–0.242)	0.115 (0.000–0.240)
RF	0.630 (0.541–0.726)	0.938 (0.924–0.952)	0.119 (0.028–0.222)	0.135 (0.031–0.256)	0.966 (0.954–0.976)	0.550 (0.499–0.611)	0.095 (−0.002–0.203)	0.127 (0.029–0.226)
GBDT	0.627 (0.543–0.722)	0.865 (0.845–0.886)	0.062 (0.024–0.109)	0.216 (0.086–0.364)	0.887 (0.867–0.906)	0.552 (0.488–0.626)	0.058 (−0.014–0.141)	0.096 (0.038–0.164)
XGB	0.622 (0.535–0.711)	0.849 (0.828–0.871)	0.060 (0.025–0.105)	0.243 (0.106–0.391)	0.870 (0.850–0.890)	0.556 (0.489–0.630)	0.059 (−0.012–0.137)	0.096 (0.041–0.162)
NaiveBayes	0.621 (0.513–0.718)	0.104 (0.087–0.122)	0.033 (0.023–0.044)	0.919 (0.824–1.000)	0.076 (0.060–0.094)	0.497 (0.449–0.537)	−0.004 (−0.067–0.049)	0.063 (0.045–0.083)
LGBM	0.615 (0.527–0.705)	0.934 (0.919–0.948)	0.089 (0.020–0.174)	0.108 (0.026–0.214)	0.962 (0.949–0.974)	0.535 (0.492–0.588)	0.064 (−0.014‐0.157)	0.098 (0.024–0.186)
KNN	0.613 (0.523–0.700)	0.668 (0.641–0.695)	0.039 (0.021–0.059)	0.378 (0.229–0.541)	0.677 (0.651–0.705)	0.528 (0.453–0.607)	0.021 (−0.035–0.083)	0.070 (0.038–0.106)
MLP	0.611 (0.514–0.710)	0.937 (0.923–0.951)	0.077 (0.000–0.172)	0.081 (0.000–0.188)	0.967 (0.956–0.977)	0.524 (0.485–0.575)	0.047 (−0.030–0.141)	0.079 (0.000–0.172)
SVM	0.582 (0.495–0.672)	0.920 (0.904–0.936)	0.067 (0.016–0.140)	0.108 (0.025–0.219)	0.948 (0.936–0.961)	0.528 (0.486–0.584)	0.045 (−0.022–0.133)	0.082 (0.020–0.163)

*Note:* SMOTE was applied only to the training set and was not applied to the test set. The 95% confidence intervals (CIs) were estimated using bootstrap resampling of the test set predictions. AUC, area under the receiver operating characteristic curve; CI, confidence interval; Accuracy, Precision/positive predictive value (PPV), Sensitivity, Specificity, Balanced accuracy and Matthews correlation coefficient (MCC); F1 score, harmonic mean of precision and recall; NMSC, non‐melanoma skin cancer.

**FIGURE 5:**
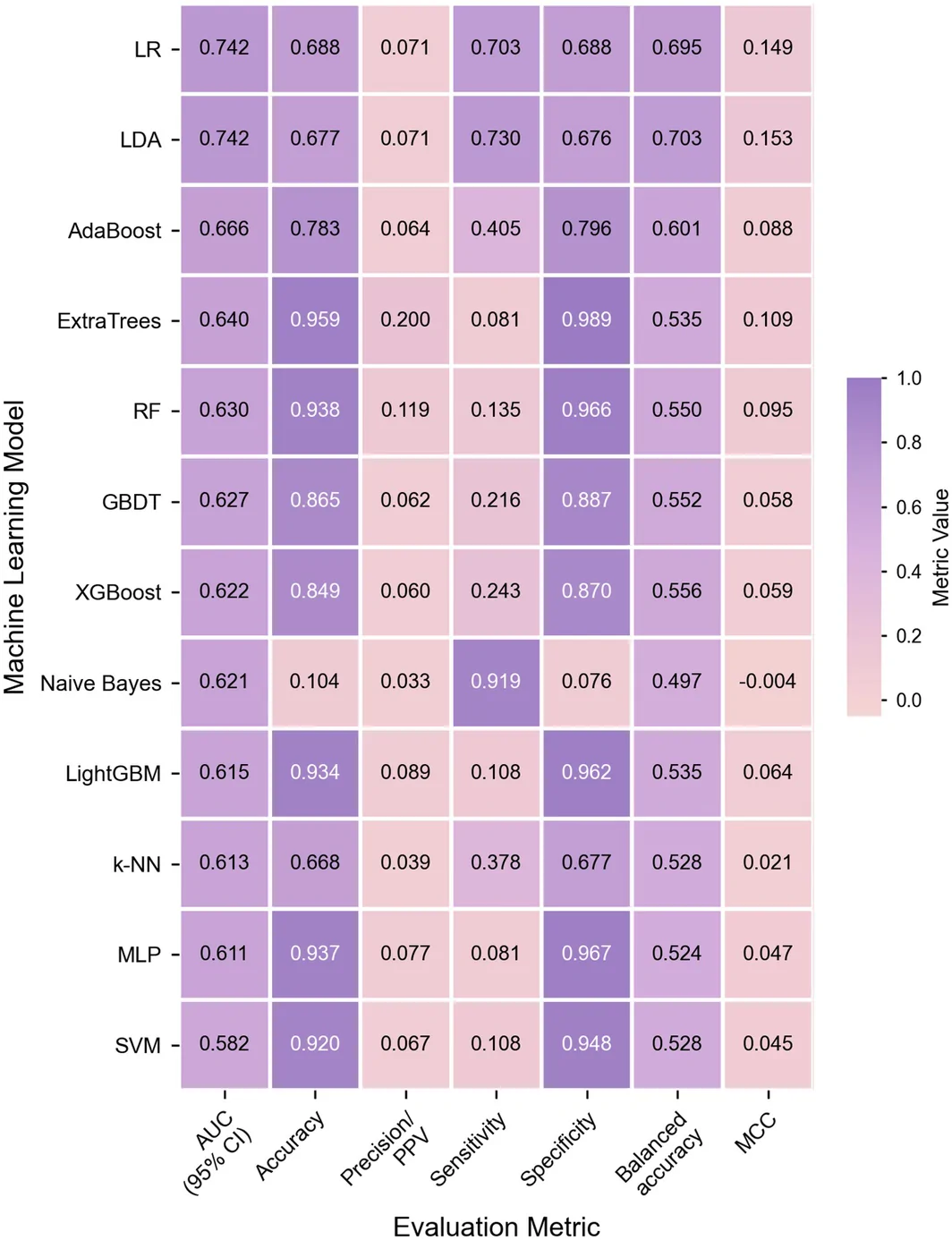
Heatmap‐based comparison of performance metrics across 12 machine learning models for final risk prediction model selection. Cell values represent point estimates from the held‐out test set, with color intensity indicating the relative metric value.

**FIGURE 6:**
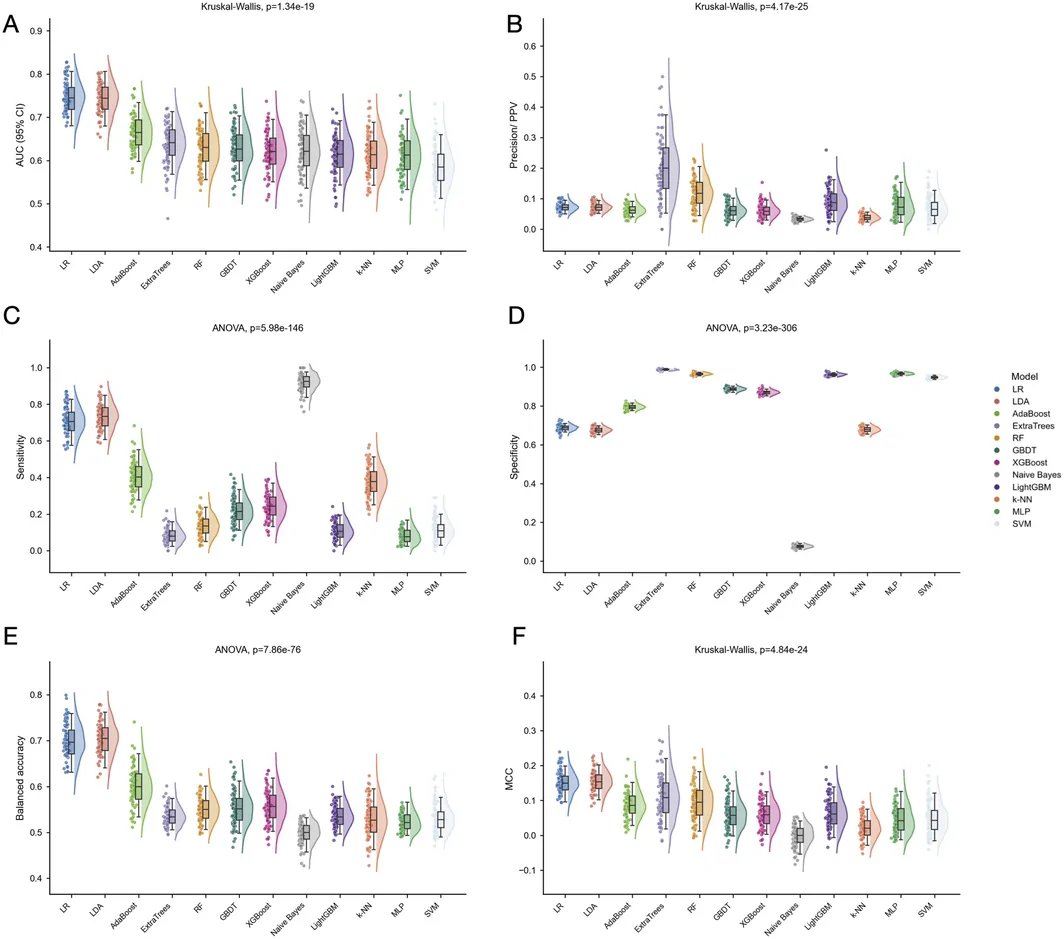
Bootstrap‐based distribution of key performance metrics across 12 machine learning models. Raincloud plots show the bootstrap distributions of six clinically relevant model performance metrics in the held‐out test set: (A) AUC (B) Precision/PPV (C) Sensitivity (D) Specificity (E) Balanced accuracy and (F) MCC. Each plot combines individual bootstrap estimates, kernel density distributions and boxplots to display model‐level variability. Omnibus *p‐*value shown above each panel compares performance across the 12 models using the indicated statistical test.

ROC curves further showed that LDA maintained a relatively stable true‐positive rate across different classification thresholds (Figure 7A). In addition, the calibration curves indicated that the probabilities predicted by the LDA model were generally consistent with the observed outcomes (Figure 7B). Decision curve analysis showed that LDA provided a higher net benefit than the other models across a broad range of threshold probabilities (Figure 7C). Therefore, LDA was selected as the final screening‐oriented prediction model because it provided the most favorable balance between AUC, sensitivity, balanced accuracy, and MCC among the candidate models.

**FIGURE 7:**
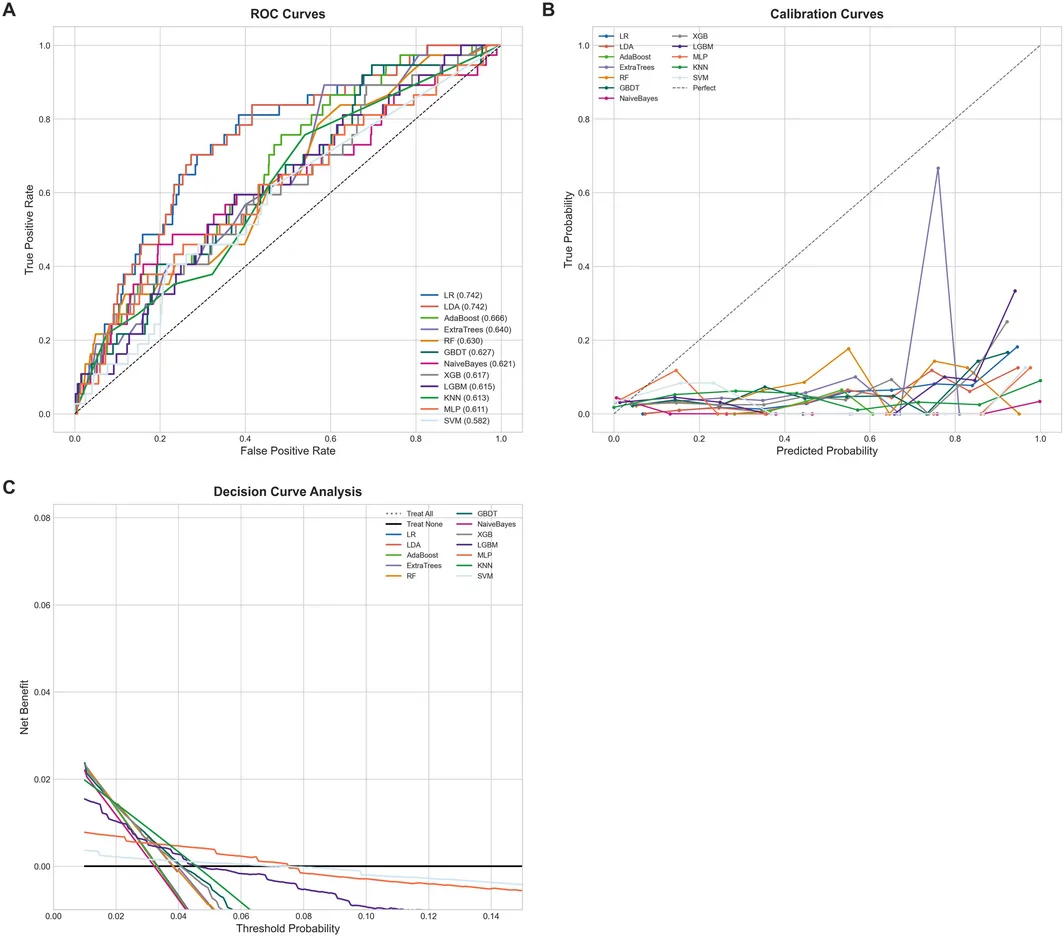
Comparative performance evaluation of 12 machine learning models for predicting non‐melanoma skin cancer risk. (A) Receiver operating characteristic curves comparing the discriminative ability of all models, with AUC values indicated for each. (B) Calibration curves assessing the agreement between model‐predicted probabilities and observed outcomes. (C) Decision curve analysis illustrating the net clinical benefit of each model across a range of threshold probabilities.

### 3.5 Global Interpretability and Feature Importance Analysis Using SHAP

We performed a SHAP‐based feature importance analysis to interpret the optimal LDA model (Figure 8). Age was the most influential predictor, with a mean absolute SHAP value of 0.71. Higher age values were consistently associated with an increased predicted risk of NMSC, which is consistent with the established epidemiological relationship between advancing age and skin cancer incidence (Wehner et al. 2018; Fania et al. 2020; Stratigos et al. 2020). Among the dietary variables, luteolin (mean absolute SHAP value = 0.49), total flavones (SHAP value = 0.44), caffeine (SHAP value = 0.36), zinc (SHAP value = 0.32), and niacin (SHAP value = 0.29) were the five most important dietary predictors (Figure 8B).

**FIGURE 8:**
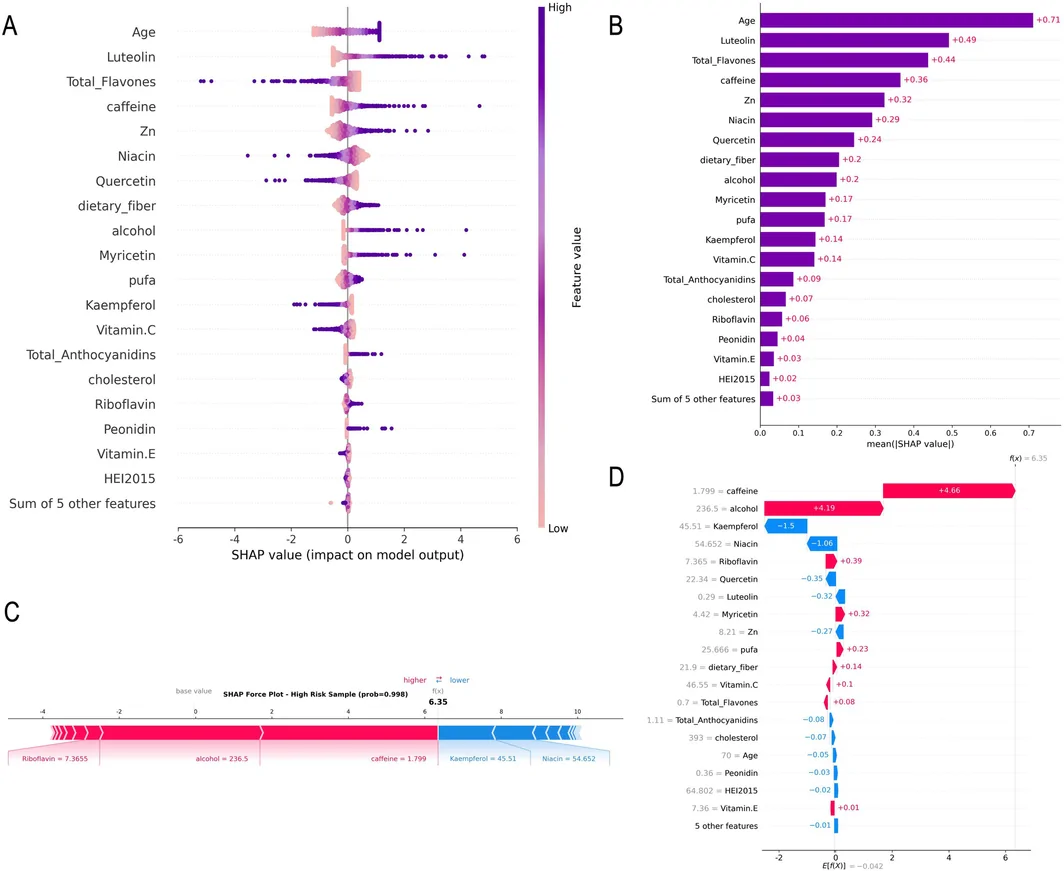
SHapley Additive exPlanations (SHAP)‐based interpretation of the optimal LDA model. (A) Beeswarm summary plot displaying the distribution and magnitude of SHAP values for all features. (B) Feature importance plot ranked by mean absolute SHAP values. (C) SHAP force plot for a representative high‐risk participant. (D) SHAP waterfall plot for a representative high‐risk participant.

The beeswarm plot (Figure 8A) showed that higher values of luteolin, caffeine, Zn, and alcohol generally contributed positively to the model output, indicating a higher predicted NMSC risk at elevated levels. In contrast, total flavones, niacin, quercetin, and kaempferol showed predominantly negative SHAP values, suggesting that higher intake levels may be associated with a lower predicted risk. Individual‐level SHAP analysis for a representative high‐risk participant with a predicted probability of 0.998 is shown in Figure 8C,D. For this participant, caffeine (SHAP value = +4.66) and alcohol (SHAP value = +4.19) were the main contributors to increased predicted risk, whereas kaempferol (SHAP value = −1.50) and niacin (SHAP value = −1.06) contributed most strongly in the opposite direction. SHAP dependence plots showed the contribution of each feature to the model‐predicted NMSC risk across the observed ranges (Figure S3). Age showed a clear risk inflection at approximately 70 years, after which its SHAP contribution increased. The plots also suggested potential interaction patterns, including inverse contributions of higher selenium and niacin levels and a stronger protective contribution of quercetin at higher myricetin intake.

## 4 Discussion

The rapid aging of the global population has increased the burden of NMSC among older adults. NMSC is closely associated with cumulative UV radiation exposure, age‐related cutaneous immunosenescence, and photoimmunosuppression (Bakker et al. 2023; Tang et al. 2024; Daniels et al. 2026), which promote carcinogenesis through DNA damage, oxidative stress, inflammatory signaling, and local immune suppression (Ciążyńska et al. 2021; Salminen et al. 2022; Pająk et al. 2023). These processes may be amplified in older adults whose immune surveillance is weakened by immunosenescence and chronic low‐grade inflammation. Such pathophysiological changes may also influence dietary behaviors, leading high‐risk individuals to adopt antioxidant‐rich or photoprotective diets and limiting the direct extrapolation of findings from the general populations. In parallel, ML methods are increasingly being applied to NHANES‐derived individual nutrients and disease data, including studies on antioxidants and cancer‐related comorbidities, as well as the application of interpretable ML in dermatology or the prediction of skin diseases. We hypothesized that various dietary antioxidant‐related factors, including micronutrients, flavonoid subclasses, CDAI, and lifestyle‐related factors, may be associated with NMSC‐related predictive patterns through pathways involving cutaneous immunosenescence and redox homeostasis. These findings may help generate hypotheses for future studies on dietary patterns and prevention strategies in this high‐risk population.

This study applied 12 machine learning models to NHANES data from 2007 to 2010 and 2017 to 2018 to identify dietary antioxidant‐related determinants of NMSC in older adults. We found that older adults with NMSC exhibited a significantly higher socioeconomic status than those without NMSC, as reflected by greater proportions of individuals with higher income (PIR: 51.6% vs. 36.8%) and postsecondary education (63.1% vs. 45.9%; both *p* < 0.001), which may reflect socioeconomically mediated differences in supplement use, outdoor activity, and cumulative UV exposure. The SHAP global summary and individual waterfall plots were used to quantify the direction and magnitude of each feature's contribution to the model predictions. Age was the most significant predictor, whereas alcohol intake was positively associated with the predicted NMSC probability. Interestingly, in contrast to previous reports of protective associations for caffeine, luteolin, myricetin, and total anthocyanins (Jung et al. 2008; Conney et al. 2013; Guo et al. 2024), these compounds were positively associated with the predicted NMSC probability in older adults. These findings may reflect age‐specific metabolic alterations or residual confounding related to socioeconomic status and lifestyle factors. Conversely, total flavones, niacin, quercetin, and kaempferol were inversely associated with the predicted NMSC probability, and the interaction patterns between vitamin C and total flavones suggested a synergistic antioxidant effect, potentially through the attenuation of chronic inflammation and modulation of cutaneous immune senescence. These findings underscore the complexity of dietary antioxidant interactions and their context‐dependent roles in NMSC susceptibility among older adults.

At the molecular level, NMSC pathogenesis involves UV‐induced epigenetic alterations, keratinocyte oxidative stress, and cutaneous immunosuppression (Prasad and Katiyar 2017; Barnes et al. 2024; Oka et al. 2025; Albulescu et al. 2026), wherein cumulative UV exposure impairs antigen presentation through the downregulation of major histocompatibility complex Class I and II molecules, suppresses T‐cell infiltration (Katiyar 2007; Soontrapa et al. 2011), and activates interleukin (IL)‐10, prostaglandin E2, and blood Cluster of Differentiation 4‐positive regulatory T cells (Durao et al. 2025), collectively facilitating NMSC progression.

Zn intake was significantly higher in the NMSC group than in the control group (11.29 vs. 9.99, *p* = 0.007). Although Zn is an essential component of antioxidant defense, excessive Zn intake may impair Cu/Zn‐ superoxide dismutase‐dependent redox homeostasis by competitively inhibiting intestinal Cu absorption (Fischer et al. 1984; Oestreicher and Cousins 1985). This finding suggests that, under conditions of chronic immune dysregulation, higher Zn intake may not necessarily confer additional antioxidant protection and may instead disturb trace element balance. This interpretation is consistent with previous reports linking high‐dose Zn‐ and selenium‐containing supplementation to an increased risk of squamous cell carcinoma (Hercberg et al. 2007). Consumption of caffeinated beverages has been associated with a reduced risk of NMSC in the Singapore Chinese population (Oh et al. 2019). However, our findings indicate that caffeine intake was positively associated with the predicted risk of NMSC. This discrepancy may reflect the fact that the estimated intake during the study period was lower than the habitual coffee intake levels of 250–400 mg/day reported in the literature. In addition, broader lifestyle or dietary‐related confounding factors, as well as the fact that SHAP values reflect predictive contributions rather than causal effects, may partially explain this inconsistency. Luteolin has been reported to exert anti‐aging effects by activating SIRT1‐related pathways, reducing oxidative stress and inflammation, preserving organelle function, and enhancing autophagy in cellular, brain‐aging, and vascular‐aging models (Xing et al. 2023; Younis et al. 2024; Yu et al. 2024). However, the relevance of habitual dietary exposure should be interpreted with caution. Its antioxidant and anti‐inflammatory effects are often observed at micromolar concentrations in vitro, whereas its daily intake is estimated to be below 1 mg/day (Seelinger et al. 2008). Moreover, after ingestion and intestinal absorption, luteolin is extensively converted from its free form into metabolites such as glucuronide conjugates, resulting in relatively low overall bioavailability (Bangar et al. 2023) and limited systemic exposure following oral intake (Sarawek et al. 2008). In addition, estimated luteolin intake may be influenced by dietary context, as its content varies substantially according to food variety and cooking method (Hostetler et al. 2013). Collectively, these findings indicate that dietary antioxidants exert complex, context‐dependent effects on NMSC risk, which may shift from protective to detrimental effects under conditions of chronic inflammation and immune senescence in older adults.

In addition, our model confirmed the relevance of established dietary antioxidants for NMSC risk prediction. Flavonoids are recognized as important antioxidant and photoprotective compounds, and niacin and quercetin ranked higher than vitamin C in terms of predictive importance. Nicotinamide, the active amide form of niacin, has been shown to enhance the repair of UV‐induced DNA damage (Thompson et al. 2014) and attenuate immunosuppression induced by UVB, UVA, and solar‐simulated UV exposure (Sivapirabu et al. 2009). Furthermore, oral nicotinamide has been shown to reduce the incidence of new NMSC and actinic keratoses in high‐risk individuals (Chen et al. 2015), as well as improve cancer survival (Ying et al. 2022). Quercetin has been shown to protect against UV‐induced photoaging and inflammation through the direct targeting of protein kinase C delta and Janus kinase 2 signaling pathways, thereby suppressing matrix metalloproteinase‐1 and cyclooxygenase‐2‐mediated collagen degradation (Shin et al. 2019). Kaempferol may exert chemopreventive effects against UV‐induced skin carcinogenesis by inhibiting Src‐mediated COX‐2/AP‐1 signaling and suppressing ribosomal S6 kinase 2/mitogen‐ and stress‐activated kinase 1 (RSK2/MSK1) activation, thereby reducing tumor incidence in UV‐induced skin cancer models (Lee et al. 2010; Yao et al. 2014). Beyond skin‐related models, both plant‐based bioactive compounds have also exhibited anticancer potential in other malignancies, including reductions in cancer cell viability (Shahid et al. 2026). Vitamin C, a water‐soluble antioxidant, may also contribute to protection against UV‐induced oxidative damage by reducing reactive oxygen species accumulation and inflammatory responses in epidermal cells (Kawashima et al. 2018). These findings suggest that our model captured biologically plausible antioxidant‐related signals and effectively identified subtle nutritional patterns associated with chronic inflammation‐related skin carcinogenesis.

Previous studies have shown that machine learning, owing to its capacity for large‐scale and high‐dimensional data analysis, has been widely applied to the prediction of major public health outcomes, including cardiovascular and skin disease. Qi et al. (2025) applied multiple machine learning models, including LightGBM, to NHANES data and used SHAP analysis to evaluate the predictive contributions of dietary antioxidants to cardiovascular disease–cancer comorbidity, identifying naringenin and magnesium as key antioxidant predictors. Similarly, categorical boosting accurately stratified disease risk in a 10‐year cohort study of children with severe pneumonia (Cui et al. 2025).

In the context of skin disease, hybrid machine learning models combining SVM and XGBoost have demonstrated superior performance in skin disease detection, achieving an accuracy of up to 99.26%, with SHAP and Local Interpretable Model‐agnostic Explanations applied to enhance interpretability and clinician trust in model outputs (Nasir et al. 2024). In the present study, we integrated dietary antioxidant features with the baseline characteristics of older adults and applied an interpretable machine learning framework to predict subtle risk‐ and protection‐related signals associated with NMSC. Based on a comprehensive evaluation of model performance, although ExtraTrees and RF showed higher overall accuracy, their relatively limited discrimination and clinical decision‐making value made them less suitable for screening‐oriented cancer risk prediction. LDA achieved the best overall balance between sensitivity, specificity, AUC, balanced accuracy, and MCC. Therefore, LDA was ultimately selected as the final screening‐oriented prediction model because it provided a more robust balance between NMSC case identification and reduced missed detection, which is particularly important for early cancer risk screening.

However, this study had several limitations. First, the NMSC diagnosis and related variables were based on self‐reported NHANES interview data, which may have introduced recall and information bias, and the questionnaire does not provide histopathological confirmation and cannot be used as a diagnostic model for clinically diagnosing NMSC or differentiating specific NMSC subtypes. Second, the cross‐sectional design precludes causal inference and may be affected by reverse causation. For example, the observed risk‐increasing signals for luteolin and other antioxidants may reflect disease‐related dietary changes rather than harmful biological effects. Third, the study survey cycles may not fully reflect current dietary patterns, supplement use, dermatologic screening or clinical management of NMSC. Finally, the absence of key UV‐related risk factors in the NHANES dataset represents a major limitation and may have biased the observed dietary antioxidant‐related predictive associations in either direction, as these features may partly reflect differences in UV exposure, sun‐protection behavior, or post‐diagnosis dietary changes rather than independent etiological effects. Although age, race, socioeconomic status, smoking, alcohol use, BMI, and other available demographic and lifestyle variables were incorporated into the prediction framework, these factors cannot fully account for individual differences in lifetime UV exposure, occupational or recreational sun exposure, sunscreen use.

Future studies should validate these findings using larger longitudinal cohorts across diverse populations and geographic regions. The incorporation of UV exposure‐related variables, genetic information, and biomarker‐based assessments may further improve causal interpretability of the model.

## 5 Conclusion

In conclusion, this cross‐sectional NHANES‐based study developed an interpretable machine learning model to identify dietary antioxidant‐related predictors of prevalent self‐reported NMSC among older adults. LDA showed the discrimination and robust performance for predicting dietary antioxidant‐related NMSC risk in older adults. SHAP analysis revealed unexpected associations between dietary nutrient profile factors and NMSC risk in the context of skin immune senescence. Caffeine, individual flavonoids, and dietary minerals may have context‐dependent effects, with high intake or supplement‐related exposure potentially serving as subtle risk factors. These findings support the promotion of balanced dietary patterns rather than excessive intake of isolated antioxidants in NMSC prevention and management strategies.

## Author Contributions

**Bo Han Yu:** data curation, conceptualization, methodology, software, formal analysis, validation, writing – original draft, visualization, investigation. **Ji Young Kang:** validation, methodology, conceptualization. **Kyung Eun Kim:** writing – review and editing, conceptualization, methodology, validation, supervision, resources, investigation, funding acquisition.

## Funding

The authors have nothing to report.

## Ethics Statement

The current study was supported by the Ethics Review Board of the U.S. National Center for Health Statistics, and written informed consents were obtained from all participants of the NAHNES survey.

## Consent

All participants provided written informed consent.

## Conflicts of Interest

The authors declare no conflicts of interest.

## Supporting information


**Figure S1:** Cross‐validation curve of the LASSO logistic regression model.
**Figure S2:** Pearson correlation heatmap of candidate variables before multicollinearity filtering.
**Figure S3:** SHAP dependency graph.
**Table S1:** Detailed calculation methods for dietary quality indices.
**Table S2:** Missingness PreImputation.
**Table S3:** Pearson correlation analysis.

## Data Availability

The datasets generated and/or analyzed during the current study are freely available on the NHANES website (https://www.cdc.gov/nchs/nhanes/). Data will be made available to the public after publication of study findings upon reasonable request from the corresponding author.
