# Supplementary material for: From Nutritional Epidemiology to Clinical Prediction: Interpretable Machine Learning Reveals Subtle Associations Between Dietary Antioxidant Profiles and Non‐Melanoma Skin Cancer in Older Adults
Source: Food Sci Nutr. 2026 Oct 20;14(10):e72458. doi: 10.1002/fsn3.72458 (PMC13681025; doi:10.1002/fsn3.72458)
Supplement: Supplementary file 1 [file FSN3-14-e72458-s001.docx]

**Supplementary Material**

**SUPPLEMENTARY FIGURE S1**. Cross-validation curve of the LASSO logistic regression model.

**SUPPLEMENTARY FIGURE S2.** Pearson correlation heatmap of candidate variables before multicollinearity filtering.

**SUPPLEMENTARY FIGURE S3**. SHAP Dependency Graph.

**SUPPLEMENTARY TABLE S1.** Detailed Calculation Methods for Dietary Quality Indices.

**SUPPLEMENTARY TABLE S2.** Missingness PreImputation.

**SUPPLEMENTARY TABLE S3.** Pearson correlation analysis.

**SUPPLEMENTARY FIGURE S1** | Cross-validation curve of the LASSO logistic regression model.

The dotted vertical lines indicate the optimal λ values based on the minimum criteria (λmin) and the 1-standard error criteria (λ1se).

**SUPPLEMENTARY FIGURE S2** | Pearson correlation heatmap of candidate variables before multicollinearity filtering.

Pearson correlation analysis was performed to assess collinearity among candidate variables. Variables with an absolute correlation coefficient |r| > 0.70 were considered highly collinear and were excluded from subsequent analyses to reduce multicollinearity. Color intensity represents the magnitude and direction of pairwise correlation coefficients.

**SUPPLEMENTARY FIGURE S3	|**	SHAP Dependency Graph.

Positive SHAP values indicate an increased contribution to the predicted NMSC risk, while negative SHAP values indicate a helpful reduction in the predicted risk.

**SUPPLEMENTARY TABLE S1 |** Detailed Calculation Methods for Dietary Quality Indices.

1. Composite Dietary Antioxidant Index (CDAI)

| **Component** | **Details** |
| --- | --- |
| Formula | CDAI = Σ(xi - μi)/Si |
| Variables | xi = daily intake of each antioxidant for individual i<br>μi = sex-specific mean intake for each antioxidant<br>Si = sex-specific standard deviation for each antioxidant |
| Antioxidant Components | Vitamin A (μg RE/day), Vitamin C (mg/day), Vitamin E (mg/day), Zinc (mg/day), Selenium (μg/day), Carotenoids (μg/day) - sum of α-carotene, β-carotene, and carotene RE |
| Interpretation | Higher scores = greater dietary antioxidant capacity<br>Standardized: mean = 0, SD = 1 <br>Positive scores = above-average antioxidant intake |

2. Dietary Inflammatory Index (DII)

| **Step** | **Process** | **Formula/Description** |
| --- | --- | --- |
| 1 | Standardization | Z-score = (Individual intake - Global mean) / Global standard deviation |
| 2 | Percentile conversion | Z-scores converted to percentile ranks |
| 3 | Centering | Centered percentile = (Percentile × 2) - 1 |
| 4 | Inflammatory scoring | Each centered percentile × inflammatory effect score |
| 5 | Summation | Sum all food parameter-specific DII scores |
| Food Parameters | 45 total parameters | Macronutrients, micronutrients, flavonoids, individual foods |
| Score Range | Theoretical range | -8.87 to +7.98 |
| Interpretation | Negative scores | Anti-inflammatory diets |
|  | Positive scores | Pro-inflammatory diets |

3.Oxidative Balance Score (OBS)

| Component Category | Components | Scoring Direction |
| --- | --- | --- |
| Dietary Antioxidants | Vitamin A, Vitamin C, Vitamin E, α-carotene, β-carotene, lycopene, Folate, selenium, zinc, Flavonoids (when available) | Higher intake = higher score |
| Dietary Pro-oxidants | Iron, Polyunsaturated fatty acids, Total fat | Lower intake = higher score |
| Lifestyle Antioxidants | Physical activity | Higher activity = higher score |
| Lifestyle Pro-oxidants | Smoking status, Alcohol consumption | Non-smokers = highest score  Lower consumption = higher score |

OBS Scoring Method

| Component Type | Categorization | Scoring |
| --- | --- | --- |
| Antioxidants | Tertiles/Quartiles | Highest tertile/quartile = highest score |
| Pro-oxidants | Tertiles/Quartiles | Lowest tertile/quartile = highest score |
| Final Score | Sum of all components | Higher OBS = greater antioxidant balance |

**SUPPLEMENTARY TABLE S2 |** Missingness PreImputation.

| Variable | Missing_n | Missing_percent |
| --- | --- | --- |
| Alcohol | 577 | 15.473317243228749 |
| caffeine | 221 | 5.926521855725396 |
| cotinine_ng.ml | 159 | 4.263877715205148 |
| cholesterol | 109 | 2.923035666398498 |
| pufa | 103 | 2.7621346205417 |
| mufa | 58 | 1.5553767766157145 |
| Vitamin D | 46 | 1.2335746849021185 |
| Education | 11 | 0.2949852507374631 |
| Stroke | 9 | 0.2413515687851971 |
| n6_fatty_acids | 8 | 0.2145347278090641 |
| carbohydrates | 6 | 0.16090104585679807 |
| protein | 6 | 0.16090104585679807 |
| Thiamin | 3 | 0.08045052292839903 |
| Hyperlipidemia | 2 | 0.053633681952266025 |
| Marital | 2 | 0.053633681952266025 |
| CVD | 1 | 0.026816840976133013 |

Number and percentage of missing values for each candidate feature before missForest imputation. Missingness was generally low to moderate across the analytic variables, with no included feature exceeding 20% missingness.

**SUPPLEMENTARY TABLE S3 |** Pearson correlation analysis.

| Variable1 | Variable2 | Correlation coefficient |
| --- | --- | --- |
| Quercetin | Total_Flavonols | 0.9615562 |
| Total_Flavonols | Kaempferol | 0.8479813 |
| Total_Flavonols | Total_Sum_of_all_29_flavonoids | 0.8415569 |
| Total_Sum_of_all_29_flavonoids | Myricetin | 0.8330061 |
| Total_Flavonols | Myricetin | 0.8137459 |
| Vitamin.B6 | Niacin | 0.8045712 |
| Quercetin | Total_Sum_of_all_29_flavonoids | 0.7806128 |
| Total_Sum_of_all_29_flavonoids | Kaempferol | 0.7717271 |
| Riboflavin | Thiamin | 0.7638702 |
| Quercetin | Myricetin | 0.7436049 |
| Thiamin | Niacin | 0.7337240 |
| Cu | Vitamin.B12 | 0.7161987 |
| Cu | Vitamin.A | 0.7033614 |

Pairwise Pearson correlation coefficients among highly correlated variables identified before feature selection. To minimize multicollinearity, redundant variables were removed prior to machine learning modeling, and only relatively independent features were retained for downstream analyses.
